# Multidrug resistance genes screening of pancreatic ductal adenocarcinoma based on sensitivity profile to chemotherapeutic drugs

**DOI:** 10.1186/s12935-022-02785-7

**Published:** 2022-12-01

**Authors:** Bangbo Zhao, Cheng Qin, Zeru Li, Yuanyang Wang, Tianhao Li, Hongtao Cao, Xiaoying Yang, Tianyu Li, Weibin Wang

**Affiliations:** Department of General Surgery, State Key Laboratory of Complex Severe and Rare Diseases, Peking Union Medical College Hospital, Chinese Academy of Medical Sciences and Peking Union Medical College, Beijing, 100730 China

**Keywords:** Pancreatic ductal adenocarcinoma, Chemoresistance, Multidrug resistance genes, Extracellular matrix

## Abstract

**Backgrounds:**

Pancreatic ductal adenocarcinoma (PDAC) is one of the most lethal cancer types and chemotherapeutic drug resistance is a stumbling block in improving the overall survival of PDAC patients. The nature of specific drug resistant subpopulation within pancreatic ductal adenocarcinoma is believed to be partly attributed to epithelial-mesenchymal transition (EMT) and cell stemness. Various PDAC cell lines show various degrees of resistance to chemotherapeutic agents including gemcitabine (GEM) and 5-fluorouracil (5-FU). In-depth understanding of drug resistance mechanisms and profile heterogeneities could lead to the development of novel and precise therapeutic strategies for addressing the chemo-resistant dilemma in PDAC patients.

**Methods:**

Cytotoxicity assays were performed by CCK8 in ten common PDAC cell lines including AsPC-1, BxPC-3, CAPAN-1, CFPAC, HPAFII, MIA PaCa-2, PANC-1, Patu-8988, SW1990 and T3M4. RNA-seq data of the ten cell lines were downloaded from Cancer Cell Line Encyclopedia (CCLE) database and subsequently analyzed for differentially expressed genes (DEGs). Based on first-line chemotherapy regimens of PDAC, DEGs between resistant and sensitive cell lines were validated by qRT-PCR. Enriched pathways of differentially expressed genes between the resistant and sensitive cell lines were acquired by Metascape database.

**Results:**

We found that the top two toxic drugs for PDAC cell lines were paclitaxel (PTX) and GEM. Among the ten PDAC cell lines, SW1990 was the most resistant PDAC cell line with the highest IC50 levels for three drugs, while MIA PaCa-2 and BxPC-3 were the most sensitive PDAC cell lines. Differential expression analysis revealed the highest number of DEGs associated with cisplatin (CIS) sensitivity up to 642 genes, of which 181 genes were upregulated and 461 genes were downregulated in CIS-resistant cell lines. The least number of DEGs are associated with GEM sensitivity, of which 37 genes were highly expressed in GEM-resistant PDAC cell lines and 25 genes were lowly expressed. Enrichment analysis of the DEGs revealed that pathways associated with drug resistance were mainly extracellular matrix and cell–cell junction related pathways.

**Conclusions:**

PDAC cell lines showed diverse sensitivities to commonly used chemotherapeutic agents, which was caused by differential gene expression between the resistant and sensitive cell lines. The heterogeneity and its associated genes were enriched in extracellular matrix and cell–cell junction related pathways. Our study first portrayed the sensitivity profile to chemotherapeutic drugs of PDAC, which would benefit the chemoresistance mechanism study by reemphasizing the vital role of extracellular matrix and cell–cell junction related pathways and helping the selection of suitable PDAC cell lines.

**Supplementary Information:**

The online version contains supplementary material available at 10.1186/s12935-022-02785-7.

## Background

PDAC is currently the fourth leading cause of cancer-related death in the USA, with an increasing incidence and poor outcome and constitutes one of the most lethal of the common malignancies with a poor five-year survival rate below 11% [1]. Despite advances in treatment strategies (including surgery, chemotherapy, radiotherapy and targeted therapies) in recent years, the survival rate of PDAC patients still remains a Gordian knot. Until now, the sole potentially curative treatment means of PDAC is surgical resection. However, for patients who have no chance to undergo surgery, the current standard regimen is chemotherapy, the first-line drugs of which include GEM, 5-FU, PTX, irinotecan (IRI) and CIS. All of the above drugs have shown unsatisfied results for which the main reason is the resistance of tumor cells to chemotherapeutic agents [2].

Chemotherapeutic regimens of PDAC are often palliative rather than curative, which means insensitivity and toxicity restrict the effectiveness of these drugs thus resulting in a very marginal improvement in the survival rate of PDAC patients with advanced disease [3]. The main cause of failure in chemotherapy is drug resistance. Drug resistance is divided into intrinsic resistance and acquired resistance in principle. For patients with intrinsic resistance, chemotherapy is ineffective from the outset, while in contrast, acquired resistance is gradually formed when tumor cells are being exposed to anti-cancer drugs and subsequently causing recurrence and metastasis [4]. Investigations into the mechanisms underlying chemoresistance were made over the past decades, but much is still unclear about exactly how it occurs or develops. Moreover, studies published have shown that the common reasons for the acquisition of drug resistance include gene mutations (such as KRAS, CCND1), altered signaling pathways (such as EMT phenotype, fibroblast growth factor receptor, cell–cell junction) [5, 6]. In clinical practice, chemotherapy resistance in PDAC patients shows significant individual heterogeneity, which reflects possible individual differences in response of cancer cells to chemotherapeutic drugs. Hitherto several specific PDAC cell lines have been built up through years with in-depth study of PDAC and the mature PDAC cell lines can reflect the heterogeneity of subpopulations within tumors of PDAC patients to some extent. Therefore, understanding sensitivities of different PDAC cell lines to different chemotherapeutic drugs and related genes and pathways can provide theoretical basis for reversing chemoresistance and precise guidance for individualized treatment of PDAC patients.

Here, we characterized the effects of five conventional PDAC chemotherapeutic drugs on ten human PDAC cell lines to determine the subgroups between chemotherapeutic sensitivity and resistance, and constructed the profile of chemotherapeutic sensitivity of PDAC cell lines. Then DEGs and their associated pathways between drug-resistant and sensitive cell lines were analyzed and enriched with the RNA-seq data from public databases. In addition, PDAC cells co-culture with pancreatic stellate cells (PSCs) were performed to verify the role extracellular pathways on drug resistance. Our results revealed abundance of DEGs associated with multidrug resistance not reported before providing hope for deliberately dealing with chemoresistance of PDAC.

## Methods

### Cell lines and culture condition

Ten common PDAC cell lines were used in this article, including AsAC-1, BxPC-3, CAPAN-1, CFPAC, HPAFII, MIA PaCa-2, PANC-1, Patu-8988, SW1990 and T3M4. T3M4 were generously provided by Dr. Chengcheng Wang (Peking Union Medical College Hospital, Beijing, China) and the others were all purchased from American Type Culture Collection (ATCC). All the cell lines were validated by detecting their specific short tandem repeats (sanger sequencing, Tsingke,Inc.). AsPC-1 and SW1990 were cultured in RPMI-1640 modified medium (Hyclone). BxPC-3 was cultured in RPMI (Corning). CAPAN-1 and CFPAC were cultured in Iscove's Modified Dubecco's Medium (IMDM, Hyclone). HPAF-II, MIA PaCa-2, PANC-1, Patu-8988 and T3M4 were cultured in Dulbecco's Modified Eagle Medium (DMEM)/High Glucose (Hyclone). The culture medium of CAPAN-1 was added with 20% fetal bovine serum (FBS) and the culture medium of the other cell lines were added with 10% FBS.

### Reagents

All five chemotherapeutic drugs, GEM (Lilly, USA), 5-FU (Kingyork, China), PTX (Peking Union Pharmaceutical Factory, China), IRI (Hengrui, China) and CIS (Qilu Pharmaceutical, China), were obtained from Peking Union Medical College Hospital.

### siRNA transfection

Gene-specific siRNAs and nonsense control were provided by Tsingke (Beijing, China). PDAC cells were transfected with siRNA using Lipofectamine 3000 (Invitrogen, California, USA). After the knockdown efficiency was confirmed by quantitative RT-PCR (qRT-PCR) and western blot, the cells were used for subsequent experiment. The sequences of siRNAs used were listed in Additional file 4: Table S3.

#### Indirect co-culture model of PSCs and PDAC cells

For indirect co-culture, PSC cells (1*10^5^/mL) were seeded in the upper berth of the co-culture chamber and PDAC cells (1*10^5^/mL) were seeded in the bottom of co-culture chamber. Co-culture for 48 h and RNA were extracted from PDAC cells.

### In vitro cell cytotoxicity assay and IC50 calculation

Cells were plated into 96-well plates and treated after 24 h with GEM, 5-FU, PTX, IRI and CIS. The concentration gradients of drugs were 0, 1 nM, 10 nM, 100 nM, 1 μM, 10 μM, 100 μM and 1 M. After cultured with drugs for 48 h, drug-containing culture medium was replaced by fresh medium which contained 10% CCK-8 (Dojindo, Japan). Subsequently, place the plates in incubator under 37℃ for 2 h and determine its light absorption value at 450 nm and 630 nm using an enzyme-linked immunosorbent detector (Invitrogen, Thermo Fisher Scientific, USA). The difference between absorbance values at 450 nm and 630 nm indirectly reflects the number of living cells.

Cell viability values under the drug concentration gradients were conducted non-linear fitting (inhibitor, four parameters). The 50% inhibitory concentration (IC50) values were defined as the concentration of drug that inhibited cell growth by 50% relative to the untreated control. IC50 values were calculated using GraphPad Prism 9.0 software (La Jolla, USA).

### Data source, identification of DEGs and enrichment analysis

The set of sequence-based mRNA expression data (RNA-seq data) of the ten PDAC cell lines were downloaded from CCLE database (http://portals.broadinstitute.org/ccle). The DEGs between drug resistant and sensitive PDAC cell lines were obtained by using edgeR (bioconductor.org/packages/release/bioc/) in R 3.6.0. Those genes with a 丨log2(fold change)丨 ≥ 2 and adjusted P value < 0.05 in the default Benjamini–Hochberg false discover rate (FDR) method were considered to have statistical significance, which was shown via volcano plot. We then used Metascape (https://metascape.org/) based on Gene Ontology (GO) analysis and Kyoto Encyclopedia of Genes and Genomes (KEGG) analysis to comprehensively analyze the biological functions, cellular components, molecular functions and pathways of the DEGs between resistant and sensitive PDAC cell lines. Venn diagrams were obtained by using jvenn (http://jvenn.toulouse.inra.fr/).

### RNA extraction and quantitative real-time PCR (qRT-PCR) analysis

Total RNA was extracted from PDAC cell lines using TRIzol (Invitrogen, CA, USA) and processed for reverse transcription and quantitative PCR using a Reverse Transcription System (Promega, Madison, WI, USA) and a One Step SYBR ® PrimeScript^tm^ RT-PCR Kit (Vazyme, Nanjing, China) according to the manufacturer’s instructions. The sequences of primers used in qRT-PCR were listed in Additional file 5: Table 4.

### Western blot assay

Whole cell lysates were obtained with RIPA lysis buffer (Applygen, Beijing, China) containing 1% protease and phosphatase inhibitors (Sigma–Aldrich, St. Louis, USA) on ice. The cell lysates were centrifuged at 12, 000 rpm for 15 min at 4 °C to remove undissolved impurities and collect the supernatants. The protein concentration was quantified using a BCA protein assay kit (Beyotime, Shanghai, China). Then, proteins were separated by 10% SDS-PAGE and transferred to 0.22 μm polyvinylidene fluoride (PVDF) membranes. The non-specific binding sites on the membrane were blocked with 5% milk for 1 h. After blocking, the membrane was first incubated with the primary antibody of UCP2 (11081-1-AP, Proteintech, Shanghai, China) overnight at 4 °C and then with the secondary antibody at room temperature for 1 h. Finally, super-sensitive ECL assay kit (Beyotime, Shanghai, China) was used to show the immune response.

### Statistical analysis

The RNA expression levels were analyzed by applying the unpaired parametric t-tests. Statistical analyses were performed using GraphPad Prism 9.0 (La Jolla, USA). A p value < 0.05 was considered statistically significant.

## Results

### Sensitivity profile to chemotherapeutic drugs of each PDAC cell line

**Fig. 1:**
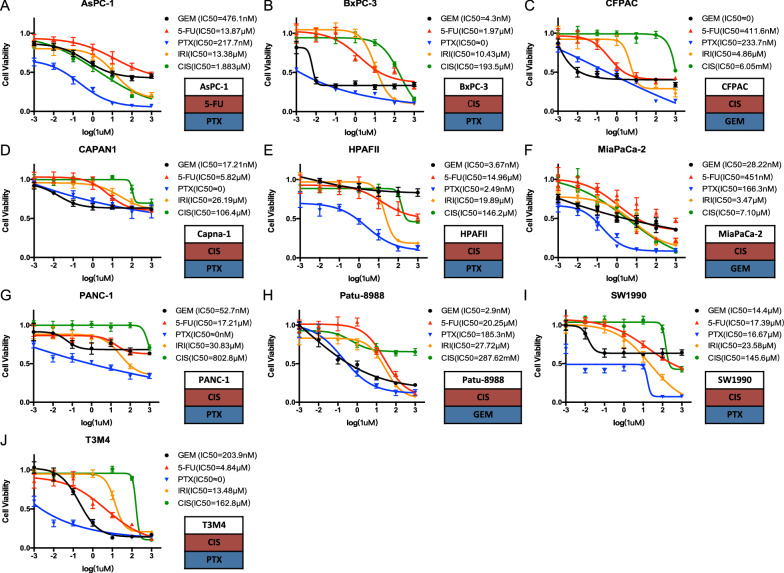
Cytotoxicity assay and IC50 of ten common pancreatic ductal adenocarcinoma (PDAC) cell lines, AsPC-1 (**A**), BxPC-3 (**B**), CFPAC (**C**), CAPAN-1 (**D**), HPAFII (**E**), MIA PaCa-2 (**F**), PANC-1 (**G**), Patu-8988 (**H**), SW1990 (**I**) and T3M4 (**J**), to five first-line chemotherapeutic drugs. The most resistant and sensitive drugs of each cell line were shown in the red and blue box respectively

We conducted cytotoxicity assay by treating ten common PDAC cell lines, namely AsPC-1, BxPC-3, CAPAN-1, CFPAC, HPAF-II, MIA PaCa-2, PANC-1, Patu-8988, SW1990 and T3M4, with five first-line chemotherapeutic drugs of PDAC, namely GEM, 5-FU, PTX, IRI and CIS. Inhibition curves based on cell inhibition ratio were shown classified by cell lines (Fig. 1). As for a single PDAC cell line, the IC50 values for each chemotherapeutic drug varied much from several nanomoles to thousands of micromoles. Nine of the ten PDAC cells were most resistant to CIS, except for AsPC-1, whose IC50 for 5-FU was much greater than that for the other four drugs. The two most toxic drugs for PDAC cell lines were PTX and GEM. CFPAC, MIA PaCa-2 and Patu-8988 were most sensitive to GEM and the other seven cell lines were most sensitive to PTX.

### Sensitivity profile of PDAC cell lines to each chemotherapeutic drug

**Fig. 2:**
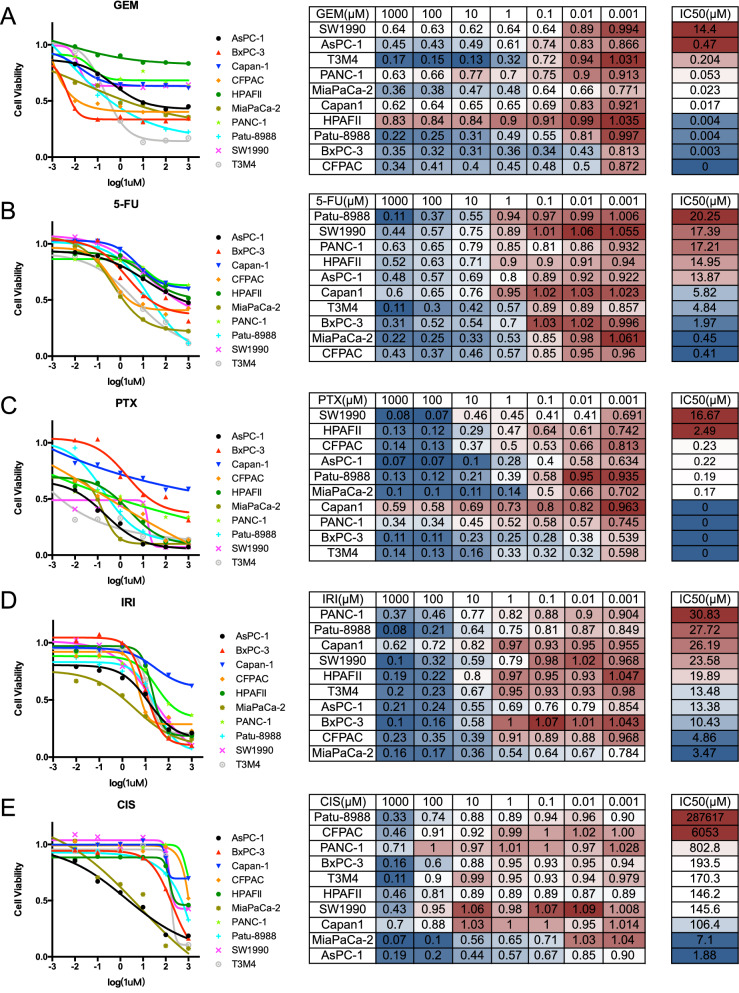
Cell viability curve and IC50 of PDAC cell lines grouped by chemotherapeutic drugs, namely GEM (**A**), 5-FU (**B**), PTX (**C**), IRI (**D**) and CIS (**E**). The color change from blue to red represented the change from 'sensitive' to 'resistant'

We then drew the inhibition curves classified by the five first-line chemotherapeutic drugs in order to compare sensitivity of the ten PDAC cell lines to a single drug visually (Fig. 2, Additional file 1: Fig. S1). For some drugs, such as 5-FU and IRI, the IC50 values of the ten PDAC cell lines had little difference and the maximum was less than 100 times of the minimum. While for the drugs including GEM, PTX and CIS, the IC50 values of cell lines varied much. PDAC cell lines were generally sensitive to GEM and PTX, as the maximal IC50 value was less than 20 μM and several of the cell lines were nearly totally sensitive to the two drugs in vitro, correlating with the fact that the two drugs could be combined into an effective and widely used regimen. Eight of the ten PDAC cell lines were extremely resistant to CIS, yet MIA PaCa-2 and AsPC-1 were sensitive to it. In this regard, genetic variants such as BRCA1/2 mutation have confirmed that PDAC is a conglomerate of multiple subtypes. Indeed, the alternations of BRCA1/2 have been noted to increase sensitivity to platinum-based chemotherapy in breast, ovarian cancer and PDAC [7–11].

**Fig. 3:**
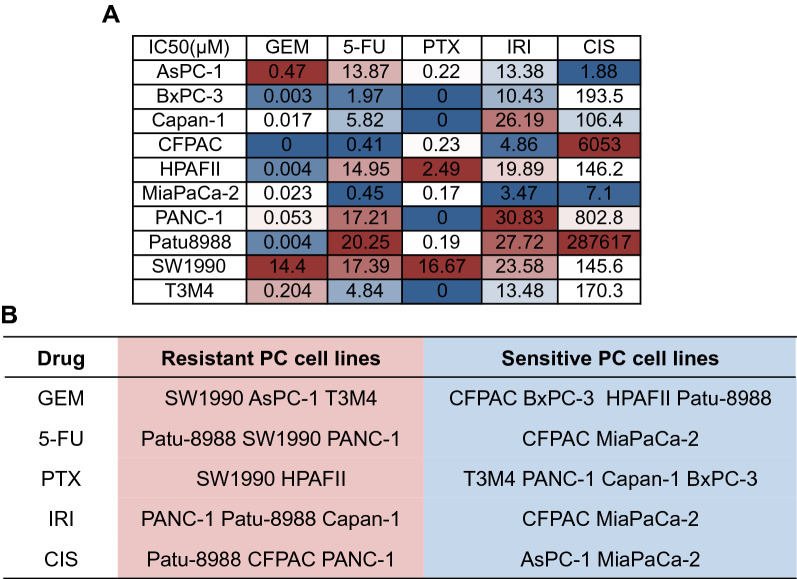
Sensitivity profile to chemotherapeutic drugs of pancreatic ductal adenocarcinoma cell lines. **A** IC50s of each PDAC cell line to each chemotherapeutic drug. **B** The most resistant and sensitive PDAC cell lines to the five chemotherapeutic drugs

IC50 values were summarized in the form of heatmap (Fig. 3A). SW1990 was the most resistant PDAC cell line as its IC50 values for three drugs, GEM, 5-FU and PTX ranked top three. MIA PaCa-2 and BxPC-3 were the most sensitive PDAC cell lines. Regimen based on GEM and regimen based on 5-FU are the two main chemotherapeutic regimens for PDAC. Out of this importance, we found that resistant PDAC cell lines to GEM were SW1990, AsPC-1 and T3M4, while the sensitive cell lines were CFPAC, BxPC-3, HPAFII and Patu-8988 (Fig. 3B). As for 5-FU, Patu-8988, SW1990 and PANC-1 were resistance to it and CFPAC and MIA PaCa-2 were sensitive.

### Identification and validation of DEGs in drug sensitive and resistant PDAC cell lines

**Fig. 4:**
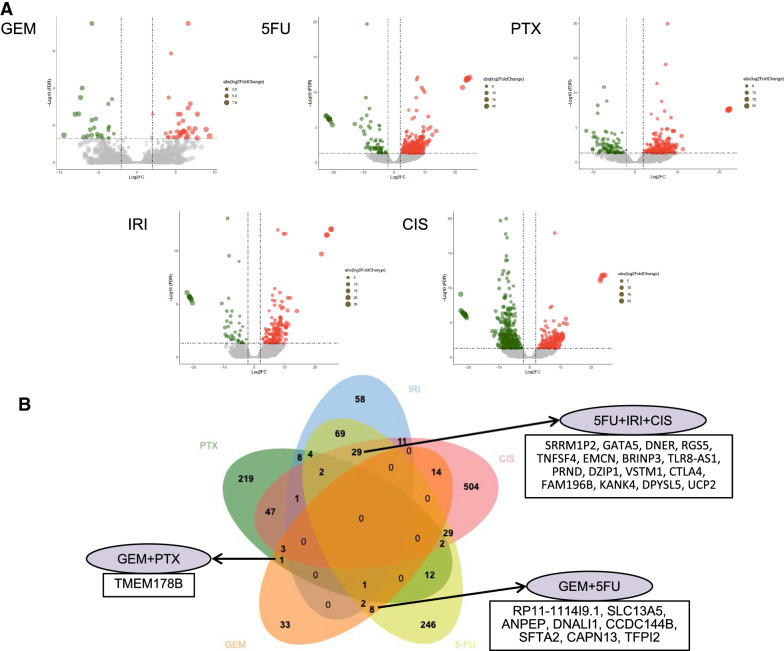
Differentially expressed genes (DEGs) between drug sensitive and resistant PDAC cell lines. **A** Volcano plots showed up-regulated genes (red plots) and down-regulated genes (green plots) in resistant PDAC cell lines of each drug. **B**. Venn diagram of DEGs between resistant and sensitive PDAC cell lines of each drug

In order to explore genes associated with drug resistance in PDAC, we compared the transcriptome data acquired from the CCLE database of the resistant and sensitive cell lines of each chemotherapeutic drug (Fig. 4A, Additional file 1: Fig. S2). DEGs associated with CIS sensitivity had the largest amount of 642 genes, in which 181 genes were up-regulated in the CIS resistant cell lines and 461 genes were down-regulated. The least DEGs were associated with GEM sensitivity, in which 37 genes expressed higher in GEM resistant PDAC cell lines and 25 genes expressed lower.

Treatment strategy of PDAC tends towards combined regimen rather than a single drug. GEM and PTX combined regimen is a widely applied first-line chemotherapeutic regimen recommended by the National Comprehensive Cancer Network (NCCN) guideline. Current clinical chemotherapy for PDAC is mostly based on 5-FU and GEM [12, 13]. Hence, to identify genes that cause chemoresistance in multidrug combination regimens, DEGs for the five drugs were intersected using a Venn diagram (Fig. 4B, Additional file 2: Table 1). The results show that several genes that have been shown to cause chemoresistance in tumors may also play an important role in multidrug combination chemotherapy for PDAC.

**Fig. 5:**
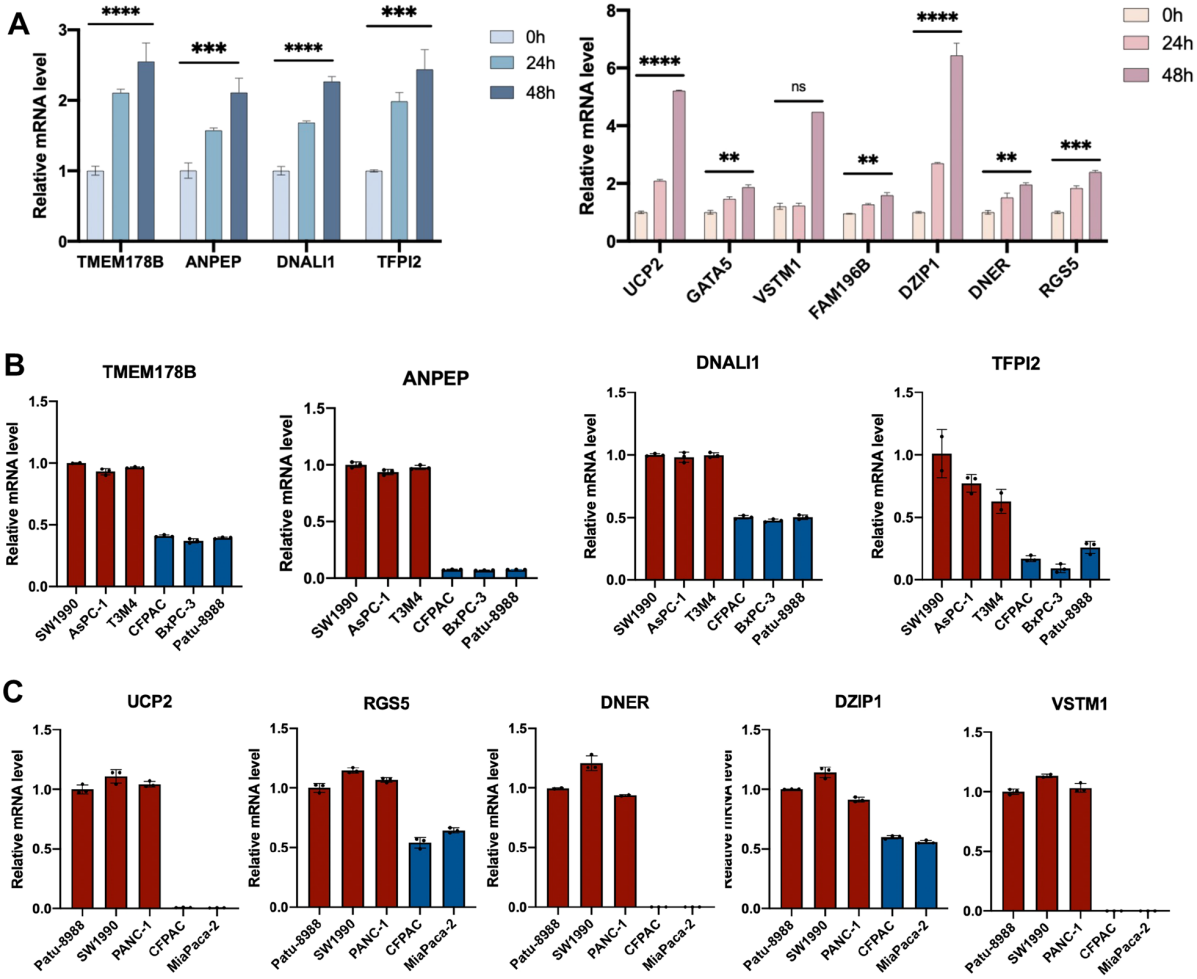
Validation of DEGs in PDAC cell lines by qRT-PCR. **A** Temporal gradient expression levels of DEGs based on GEM. **B** Expression level of DEGs based on GEM in resistant/sensitive PDAC cell lines. **C** Temporal gradient expression levels of DEGs based on 5-FU. **D** Expression level of DEGs based on 5-FU in resistant/sensitive PDAC cell lines

Several multidrug resistance associated genes were confirmed by qRT-PCR. We stimulated MIA PaCa-2 and BxPC-3 PDAC cell lines with 5-FU and GEM, respectively, and observed a time-dependent increase in the expression of drug resistance associated genes, including TMEM178B, ANPEP, DNALI1, TFPI2, UCP2, GATA5, VSTM1, FAM196B, DZIP1, DNER and RGS5 (Fig. 5A, B). Furthermore, some of the DEGs were also verified in PDAC cell lines (Fig. 5C, D). Some of the validated DEGs have been studied in tumor drug resistance. The transcription factors in GATA family have been reported to regulate tumor development. TCR signaling activates a signaling axis that includes ITK, NF-κB, and GATA-3 and promotes chemotherapy resistance in non-Hodgkin lymphomas [14]. Uncoupling protein 2 (UCP2) promotes proliferation and chemoresistance via NF-κB/β-catenin axis in gallbladder cancer[15]. To verify the accuracy of the DEGs, we selected UCP2 for knockdown in the multidrug-resistant cell line PANC-1 and found that drug resistant ability of PANC-1 was alleviated after UCP2 knocking down (Additional file 1: Fig. S3A–C).

### Enrichment analysis reveals extracellular matrix and cell–cell junction matters in PDAC chemoresistance

**Fig. 6:**
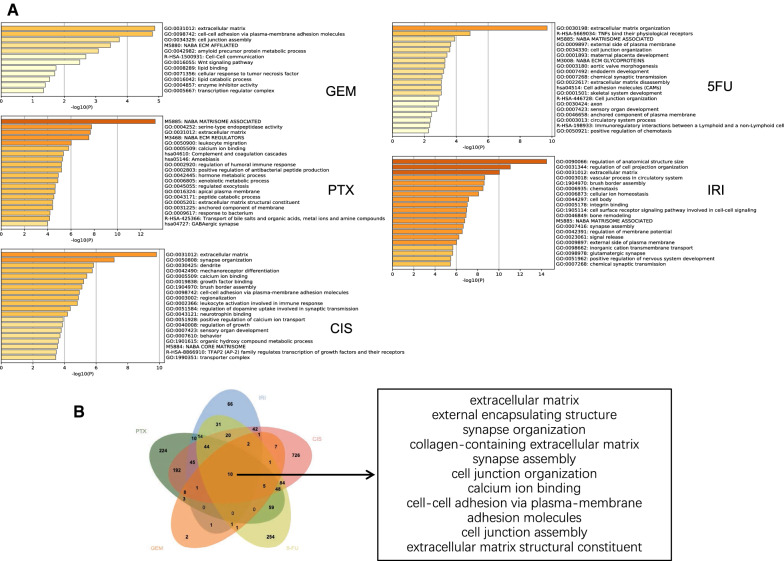
Enrichment analysis of DEGs between drug sensitive and resistant PDAC cell lines. **A** Top biological processes, cellular components, molecular functions and pathways enriched based on DEGs between drug sensitive and resistant PDAC cell lines. **B**. Venn diagram of enriched items of DEGs between resistant and sensitive PDAC cell lines of each drug

**Fig. 7:**
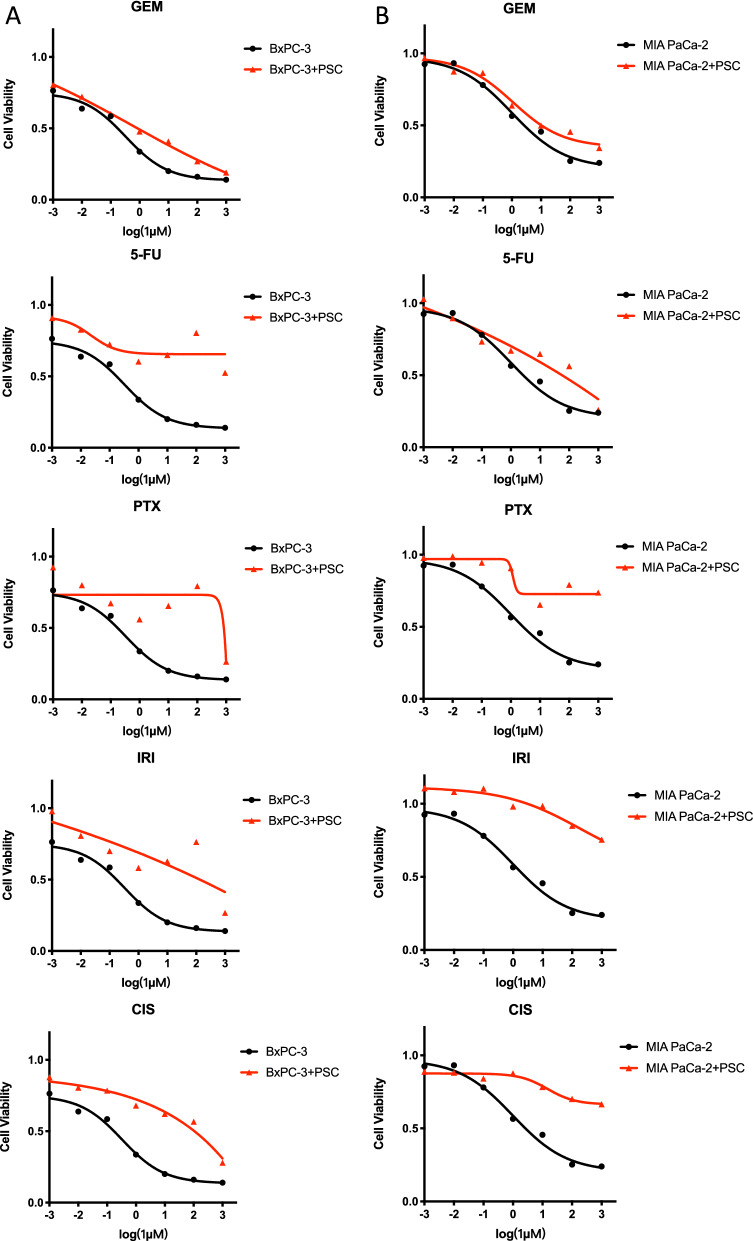
Cell viability curves of PDAC cells to five drugs (GEM, 5-FU, PTX, IRI, CIS) after co-culture with PSCs. **A**. MIA PaCa-2, **B**. BxPC-3

GO and KEGG pathway enrichment analyses were applied to discover the functions of the DEGs between drug sensitive and resistance PDAC cell lines (Fig. 6A, Additional file 3: Table 2). The DEGs were significantly enriched in biological processes associated with extracellular matrix and cell adhesion. Previous studies have proposed multiple mechanisms of drug resistance in PDAC, including abnormal gene expression, mutations, dysregulation of key signaling pathways (such as NF-κB, Akt and apoptosis-related pathways), EMT and the role of extracellular stromal cells and cancer stem cells [16]. This finding is consistent with the accepted mechanism of drug resistance in PDAC. KEGG pathway analysis revealed that DEGs are mainly involved in extracellular matrix, external encapsulating structure, collagen-containing extracellular matrix, cell junction organization, cell–cell adhesion via plasma-membrane and cell junction assembly extracellular matrix structural constituent. In addition, pathways related to synaptic structure and calcium binding are also enriched (Fig. 6B). We selected two multidrug-sensitive cell lines, MIA PaCa-2 and BxPC-3 based on the above drug resistant profile and co-culture both the two cell lines with PSC. As expected, these two cell lines were found to have increased resistant capability to all the five drugs after 48 h of co-culture (Fig. 7). This result shows a good indication of the important role of extracellular signaling pathways in drug resistance of PDAC cells.

## Discussion

Drug resistance is a main cause of poor effect of chemotherapy for PDAC patients. Repeated attempts seeking for multidrug combined regimens have failed to achieve satisfied results. Overcoming chemotherapy resistance is therefore a major challenge in prolonging the overall survival of PDAC patients. In this study, we compared the levels of multidrug resistance between different PDAC cell lines by assessing the sensitivity of 10 common PDAC cell lines to five first-line chemotherapeutic agents and combination of drugs. DEGs and associated molecular pathways between drug-resistant and sensitive cell lines were also analyzed and validated. Importantly, there was significant heterogeneity in response of different PDAC cell lines to different chemotherapeutics, and the heterogeneity among PDAC cell lines was closely related to multidrug resistance associated genes and molecular pathways. To validate the role of key genes and extracellular pathways on PDAC cell drug resistance, we knocked down UCP2, one of the screened key genes (Fig. 6) in drug-resistant cell line PANC1, and found that UCP2 knockdown resulted in reduced drug resistance of PANC1.

Global PDAC cell line chemosensitivity profile in our study unveils that various pancreatic ductal adenocarcinoma cell lines show different sensitivities to different drugs. For example, AsPC-1 is resistant to GEM but sensitive to CIS, and PANC-1 is resistant to 5-FU but sensitive to PTX. In addition, we identified multidrug-resistant cell lines SW1990, Patu-8988 and PANC-1, and multidrug-sensitive cell lines MIA PaCa-2, BxPC-3 and CFPAC. In addition, we performed multidrug co-treatment (FOLFIRINOX strategy, 5-FU + CIS + IRI) in the sensitive cell line MIA PaCa-2 and the drug-resistant cell line PANC-1 and found that heterogeneity between PDAC cell lines still persisted when response to clinically common combinations of chemotherapeutic agents (Additional file 1: Fig. S4). These results suggest a clear heterogeneity of PDAC cells in chemotherapeutic drug response. Taking advantage of the heterogeneity, we can classify PDAC cell lines into different subpopulations by drug sensitivity, helping us effectively selecting suitable cell lines for PDAC chemoresistance research in the future. By comparing the characteristics of heterogeneous PDAC cell lines, including differential expression of genes and differential activation of vital molecular pathways, we could made further understanding of the chemoresistance formation of PDAC.

To gain mechanistic insights into chemoresistance of multidrug, we analyzed DEGs and multiple pathways between drug-sensitive and resistant PDAC cell lines. Based on pathway enrichment analysis of DEGs, we found that different drugs lead to drug resistance in PDAC by different mechanisms. For example, GEM leads to resistance mainly through pathways related to extracellular matrix, cell–cell adhesion and cell junction assembly, while CIS leads to drug resistance mainly through regulation of anatomical structure size, cell projection organization, cellular ion homeostasis and other pathways. However, what is of greater interest is that all five drugs lead to drug sensitivity through pathways such as extracellular matrix, external encapsulating structure, synapse organization, collagen-containing extracellular matrix, synapse assembly, cell junction, etc. The results suggested that different chemotherapy drugs have synergistic effects in inhibiting tumor progression. To verify the role of extracellular pathways in drug resistance, we co-cultured two sensitive PDAC cell lines, MIA Paca2 and BxPC-3, with PSC and surprisingly found that both the two cell lines became more resistant after 48 h of co-culture. The implications of this finding for clinical practice lie in the use of multidrug combination regimens, where chemotherapeutic agents with different mechanisms are combined in different subgroups of patients with individual differences or at different stages of the patient's disease course. Besides, the recognition of multidrug resistance associated genes and pathways of PDAC were in favor of reversing chemoresistance by targeting the related factors.

Among the above pathways associated with drug resistance, it is worth mentioning EMT. EMT is the process by which epithelial cells lose apical-basal polarity and cell–cell adhesion, and transit to invasive mesenchymal cells [17]. As a well-studied, complex and dynamic epigenetic level reprogramming event, one of the main roles of EMT is to promote the adaptation of cancer cells to conditions in tumor microenvironment to ensure survival, thereby generating cellular heterogeneity and promoting drug resistance [18]. Cancer cells with EMT feature have a more pronounced growth advantage after drug screening and may lead to metastasis after chemotherapy [19]. Signaling pathways that promote EMT contribute to the development of cellular drug resistance. For example, the TGF-beta pathway induces EMT leading to drug resistance, and the activation of related pathways following cellular resistance leads to high TGF-beta expression and active related pathways [20, 21]. EMT-related transcription factors such as Snail, Slug and ZEB have also been reported to be associated with drug resistance of PDAC [22, 23].

Overall, our study provided the first summary of sensitivities of common PDAC cell lines to clinically used chemotherapeutic agents. The response of different cell lines to the same chemotherapeutic agent and the toxicity of different chemotherapeutic agents to the same cell line were compared in detail. The DEGs and associated pathways between resistant and sensitive cells in the same drug were analyzed. However, translating the heterogeneity of PDAC cells in terms of drug resistance into clinical dosing regimens or targeted therapy to reserve chemoresistance still requires more in-depth exploration.

## Conclusions

In this study, we identified drug heterogeneity among PDAC cell lines by assessing the sensitivity of 10 PDAC cell lines to five first-line chemotherapeutic agents. Through the analysis and enrichment of differentially expressed genes and their associated pathways between drug-resistant and sensitive cell lines, we found that drug resistance in PDAC cells is mainly caused by EMT and abnormal cell–cell junction. In addition, PDAC is typically characterized by extensive tumor-associated stroma, and several studies have confirmed that stromal cells also play a key role in the drug resistance process. Overall, we remain confident that the establishment of PDAC cell resistance profile and the dissection of related molecular biological pathways are the starting point for addressing the individual heterogeneity of drug resistance in clinical pancreatic ductal adenocarcinoma patients. We expect to ultimately achieve more effective clinical chemotherapy regimens and improved disease control of pancreatic ductal adenocarcinoma patients.

## Supplementary Information

**Additional file 1: Figure S1**. Cell viability curve and IC50 of PDAC cell lines grouped by chemotherapeutic drugs, namely GEM, 5-FU, PTX, IRI and CIS. The color change from blue to red represented the change from 'sensitive' to 'resistant'. **Figure S2**. Up- and down-regulated DEGs in resistant PDAC cell lines of each chemotherapeutic agents. (A) Up-regulated DEGs in resistant PDAC cell lines of each drug. (B) Down-regulated DEGs in resistant PDAC cell lines of each drug. **Figure S3**. Knocking down UCP2 results in reduced drug-resistant ability of PANC-1. (A) qRT-PCR to verify the efficiency of UCP2 siRNAs. (B) Western blot to verify the efficiency of UCP2 siRNAs. (C) Cell viability curve of PANC-1 after knocking down UCP2. **Figure S4**. Synergetic effects of 5-FU+IRI+CIS on sensitive cell line MIA PaCa-2 and resistant cell line PANC-1.

**Additional file 2: Table S1.** Intersection of DEGs between resistant and sensitive PC cell lines.

**Additional file 3: Table S2.** Intersection of DEGs pathway enrichment for each first-line PC chemotherapeutic drugs.

**Additional file 4: Table S3.** The sequences of siRNA used in this article.

**Additional file 5: Table S4.** The sequences of primers used in this article.

## Data Availability

The original contributions presented in the study are included in the article/ Supplementary Material. Further inquiries can be directed to the corresponding author. No additional data available.

## Abbreviations

PDAC: Pancreatic ductal adenocarcinoma
EMT: Epithelial-mesenchymal transition
GEM: Gemcitabine
5-FU: 5-Fluorouracial
PTX: Paclitaxel
CIS: Cisplatin
IRI: Irinotecan
CCLE: Cancer Cell Line Encyclopedia
DEGs: Differentially expressed genes
qRT-PCR: Quantitative real-time polymerase chain reaction
IC50: Half maximal inhibitory concentration
ATCC: American Type Culture Collection
GO: Gene ontology
KEGG: Kyoto Encyclopedia of Genes and Genomes
NCCN: National Comprehensive Cancer Network
PSCs: Pancreatic stellate cells
UCP2: Uncoupling protein 2
