# Supplementary material for: Multidrug resistance genes screening of pancreatic ductal adenocarcinoma based on sensitivity profile to chemotherapeutic drugs
Source: Cancer Cell Int. 2022 Dec 1;22:374. doi: 10.1186/s12935-022-02785-7 (PMC9714099; doi:10.1186/s12935-022-02785-7)
Supplement: Supplementary file 1 — Additional file 1: Figure S1. Cell viability curve and IC50 of PDAC cell lines grouped by chemotherapeutic drugs, namely GEM, 5-FU, PTX, IRI and CIS. The color change from blue to red represented the change from 'sensitive' to 'resistant'. Figure S2. Up- and down-regulated DEGs in resistant PDAC cell lines of each chemotherapeutic agents. (A) Up-regulated DEGs in resistant PDAC cell lines of each drug. (B) Down-regulated DEGs in resistant PDAC cell lines of each drug. Figure S3. Knocking down UCP2 results in reduced drug-resistant ability of PANC-1. (A) qRT-PCR to verify the efficiency of UCP2 siRNAs. (B) Western blot to verify the efficiency of UCP2 siRNAs. (C) Cell viability curve of PANC-1 after knocking down UCP2. Figure S4. Synergetic effects of 5-FU+IRI+CIS on sensitive cell line MIA PaCa-2 and resistant cell line PANC-1. [file 12935_2022_2785_MOESM1_ESM.docx]

Supplementary Figure 1. Cell viability curve and IC50 of PDAC cell lines grouped by chemotherapeutic drugs, namely GEM, 5-FU, PTX, IRI and CIS. The color change from blue to red represented the change from 'sensitive' to 'resistant'.

Supplementary Figure 2. Up- and down-regulated DEGs in resistant PDAC cell lines of each chemotherapeutic agents. (A) Up-regulated DEGs in resistant PDAC cell lines of each drug. (B) Down-regulated DEGs in resistant PDAC cell lines of each drug.

Supplementary Figure 3. Knocking down UCP2 results in reduced drug-resistant ability of PANC-1. (A) qRT-PCR to verify the efficiency of UCP2 siRNAs. (B) Western blot to verify the efficiency of UCP2 siRNAs. (C) Cell viability curve of PANC-1 after knocking down UCP2.

Supplementary Figure 4. Synergetic effects of 5-FU+IRI+CIS on sensitive cell line MIA PaCa-2 and resistant cell line PANC-1.
