# Supplementary material for: Multidrug resistance genes screening of pancreatic ductal adenocarcinoma based on sensitivity profile to chemotherapeutic drugs
Source: Cancer Cell Int. 2022 Dec 1;22:374. doi: 10.1186/s12935-022-02785-7 (PMC9714099; doi:10.1186/s12935-022-02785-7)
Supplement: Supplementary file 4 — Additional file 4: Table S3. The sequences of siRNA used in this article. [file 12935_2022_2785_MOESM4_ESM.docx]

**Supplementary Table 3:The sequences of siRNA used in this article.**

| **Names** | **Sequences** |
| --- | --- |
| siUCP2-NC-F | AAAA |
| siUCP2-NC-R | UUUU |
| siUCP2#1-F | GCACCGUCAAUGCCUACAA |
| siUCP2#1-R | UUGUAGGCAUUGACGGUGC |
| siUCP2#2-F | GCUAAAGUCCGGUUACAGA |
| siUCP2#2-R | UCUGUAACCGGACUUUAGC |
| siUCP2#3-F | GUCAAGACGAGAUACAUGA |
| siUCP2#3-R | UCAUGUAUCUCGUCUUGAC |
