# Supplementary material for: Multidrug resistance genes screening of pancreatic ductal adenocarcinoma based on sensitivity profile to chemotherapeutic drugs
Source: Cancer Cell Int. 2022 Dec 1;22:374. doi: 10.1186/s12935-022-02785-7 (PMC9714099; doi:10.1186/s12935-022-02785-7)
Supplement: Supplementary file 5 — Additional file 5: Table S4. The sequences of primers used in this article. [file 12935_2022_2785_MOESM5_ESM.docx]

**Supplementary Table 4:The sequences of primers used in this article.**

| **Names** | **Sequences** |
| --- | --- |
| TMEM178B-F | CCTCACACTCATCTCGGGATTCTTC |
| TMEM178B-R | TCGGGTCTGGATTTAGGGTAGTTGG |
| ANPEP-F | AGGACGCCACCTCTACCATCATC |
| ANPEP-R | GGAGAACGAGCCACCACCATAATC |
| DNALI1-F | GATTCCGCCCGCAGACTCTTTG |
| DNALI1-R | GACTTTCAGTAGCCGAGCCTTGG |
| TFPI2-F | GCAACGCCAACAATTTCTACACCTG |
| TFPI2-R | CACTGGTCGTCCACACTCACTTG |
| UCP2-F | CCTCACACTCATCTCGGGATTCTTC |
| UCP2-R | TCGGGTCTGGATTTAGGGTAGTTGG |
| GATA5-F | AGGACGCCACCTCTACCATCATC |
| GATA5-R | GGAGAACGAGCCACCACCATAATC |
| VSTM1-F | TGCATCTCCATCCTTCTCCTCTTCC |
| VSTM1-R | AATGGCTGGTTCTCTTGGTGGATTC |
| FAM196B-F | ACACCAGGCACAGAGGAACTTAAAC |
| FAM196B-R | GAACTGTGAGTCCGTGGAGATGAAG |
| DZIP1-F | GGCAGCTTCGGGAAGAACACAG |
| DZIP1-R | GCGGGCTTGGGAGAATCATCAG |
| DNER-F | ACAGGTGAAGAGTGCGACATTGAC |
| DNER-R | ATTGGAGGTGGATCTCACAGTTTGC |
| RGS5-F | AACCTGGTGGAACCTTCCCTGAG |
| RGS5-R | GCACAAAGCGAGGCAGAGAATCC |
